# Scintigraphic Assessment of Lung Perfusion and Ventilation in Patients After Pneumonectomy

**DOI:** 10.3390/jcm14248849

**Published:** 2025-12-14

**Authors:** Karina Witkiewicz, Małgorzata Edyta Wojtyś, Norbert Wójcik, Krzysztof Safranow, Jarosław Pieróg, Jacek Szulc, Tadeusz Sulikowski, Konrad Jarosz, Tomasz Grodzki, Janusz Wójcik

**Affiliations:** 1 Clinical Department of Lung Diseases, Pomeranian Medical University, Alfreda Sokołowskiego 11, 70-891 Szczecin, Poland; 2 Department of Thoracic Surgery and Transplantation, Pomeranian Medical University, Alfreda Sokołowskiego 11, 70-891 Szczecin, Poland; 3 Clinic of General, Minimally Invasive and Gastroenterological Surgery, Pomeranian Medical University, Unii Lubelskiej 1, 71-252 Szczecin, Poland; 4 Department of Biochemistry and Medical Chemistry, Pomeranian Medical University, 70-111 Szczecin, Poland; 5 Clinic of Anesthesiology and Intensive Care, Pomeranian Medical University, Unii Lubelskiej 1, 71-252 Szczecin, Poland

**Keywords:** pneumonectomy, ventilation, perfusion, V/Q ratio, scintigraphy

## Abstract

**Background/Objectives:** The physiological ventilation–perfusion ratio (V/Q) in the upper pulmonary field is >3 and in the lower pulmonary field it is <1 due to the effect of gravity when the body is in an upright position. Pneumonectomy leads to significant changes in ventilation and perfusion conditions. The aim of this study was to evaluate perfusion and ventilation after pneumonectomy complicated by pleural empyema, including the relationship between surgical outcomes, sex, and time from pneumonectomy. **Methods:** The study group included 30 patients (25 men, 5 women) who underwent pneumonectomy complicated by pleural empyema. Lung function was assessed using ventilation–perfusion scintigraphy. Twenty-one patients were assessed within 5 years after pneumonectomy and nine patients >5 years after pneumonectomy. **Results:** Average flow was 21.1% in the upper field, 47.8% in the middle field, and 30.35% in the lower field. The mean perfusion value was significantly higher in the lower field of the right lung than in the lower field of the left lung (33.35 vs. 28.05, *p* = 0.001). Average ventilation was 17.21% in the upper field, 46.73% in the middle field, and 34.28% in the lower field. The mean V/Q in the upper field was in the range of 0.81–0.87, but it reached approximately 1 (0.96–1) in the middle field and exceeded 1 (1.05–1.25) in the lower field. **Conclusions:** Pneumonectomy led to increased perfusion in the upper pulmonary field and increased ventilation in the lower pulmonary field compared to the literature for patients with the two lungs (the two-lung system), with a reversal of the V/Q between the upper and lower field.

## 1. Introduction

Respiration is a complex process in which lung function plays a crucial role. The basis for an initial examination of lung function is spirometry and plethysmography supplemented by determination of the diffusing capacity of the lungs for carbon monoxide (DLCO) [1]. Further in-depth examination of lung function is achieved using scintigraphy to measure both ventilation and perfusion functions [2,3]. This examination measures radioisotope radiation in the respiratory air (ventilation scintigraphy) and the pulmonary circulation (perfusion scintigraphy). Each process being evaluated separately and in combination allows for the assessment of regional ventilation–perfusion efficiency in individual areas of the examined lung, with determination of the ventilation–perfusion ratio (V/Q). The V/Q defines areas with normal and impaired lung function to identify the lung areas with “futile ventilation” and/or “futile perfusion”, which is important in clinical management [3,4,5,6,7].

Under physiological conditions, the right lung has greater volume than the left lung, which has to be taken into account when interpreting scintigraphy results. Therefore, perfusion and ventilation are greater in the right lung than in the left lung (50–55% vs. 45–50% for perfusion and 55% vs. 45% for ventilation, respectively) [2,3,8]. The anatomical and functional differences between the right and left lung are made more complex by the differences between the upper and lower fields of a single lung. Ventilation is 1.5-times greater of the lower lung fields than in the upper fields, and the perfusion at the base of the lung is 9-times greater than the perfusion in the apex of the lung [2,3,8].

**Figure 1:**
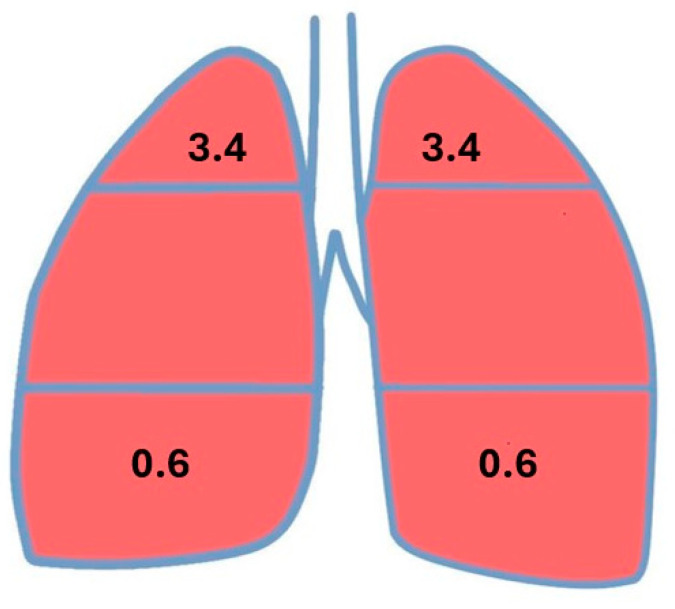
The ventilation–perfusion ratio in different lung fields under physiological conditions.

The V/Q at rest, the ratio of minute ventilation (V: ~5 L) to minute pulmonary perfusion (Q: 5.5–6 L), should not exceed 1. The mean literature value for the V/Q is assumed to be 0.85 [3,9]. This means that a V/Q value higher or lower than 0,85 gradually meets the criteria for the V/Q mismatch. However, the value differs between lung fields; it is greater in the upper fields than in the lower fields as a result of different anatomical relationships between the particular lung fields and the heart (above or below the heart level) in the vertical body position. The value of V/Q in the apex is 3.4 and at the base of the lung it is 0.6 [9] (Figure 1).

The differences in the V/Q between the apex and base of the lung are especially evident in patients after pneumonectomy. After pneumonectomy, the flow in the upper lung field is increased, reflecting the use of the flow reserve after lung resection, but the literature on this topic is rather scarce. The aim of this study was to assess the perfusion and ventilation of the single lung after pneumonectomy in an available group of patients. We also determined V/Q and analyzed the influence of operated side, sex, and time from pneumonectomy on the assessed parameters.

## 2. Materials and Methods

### 2.1. Participants

The study group consisted of 30 patients who underwent pneumonectomy in the years 1995–2018; 28 patients were operated on for oncological reasons (lung cancer) and 2 for non-oncological reasons (lung abscess and gunshot wound) (Table 1).

**Figure 2:**
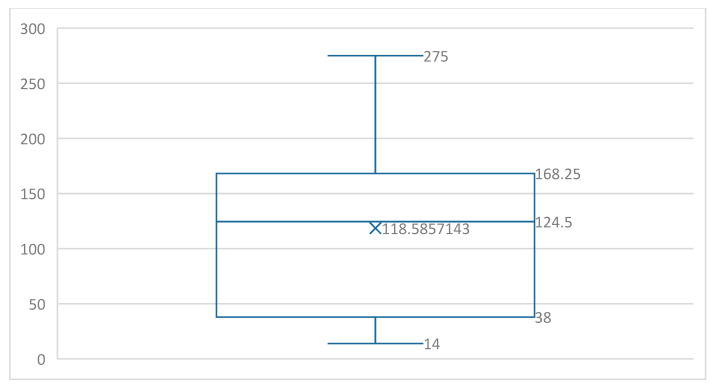
Survival of patients with lung cancer. Values are in months.

In the group of oncological patients, indication for standard pneumonectomy with mediastinal lymphdenectomy was lung cancer in hilar position. In this group of patients, 18 (64.3%) had surgery alone, 3 (10.7%) received adjuvant chemotherapy, 5 (17.8%) received adjuvant radiotherapy, and 2 (7.2%) received adjuvant radiochemotherapy. The 5-year survival in the group of oncological patients was 75% (*n* = 21) and 10-year survival was 60.7% (*n* = 17). The survival results for patients with lung cancer are presented in Figure 2. In the non-oncological group, one patient was operated on due to a non-specific lung abscess with an uncomplicated postoperative course, and another one was operated on due to gunshot damage of the lung with creation of a permanent thoracostomy.

Among the oncological patients, 25% (*n* = 7) died directly of lung cancer and 32.1% (*n* = 9) died of competing causes; 42.9% (*n* = 12) of the patients remained under observation. The duration of observation ranged from 14 to 275 months (mean 118.5 months) (Figure 2). A common characteristic of all patients in the study group was that the pneumonectomy was complicated by pleural empyema, and scintigraphic examinations were an element of their assessment. The treatment duration for pleural empyema ranged from 1.5 to 541 months (mean 43.8 months), and the time from pneumonectomy to scintigraphy ranged from 42 to 16,469 days (mean 1893 days). The methods used to treat pleural empyema were accelerated treatment in 25 patients (83.33%), passive pleural drainage in 3 patients (10%), and thoracostomy in 2 patients (6.67%). Twenty-five patients (83.33%) were assessed after treatment for pleural empyema was completed and five patients (16.67%) were assessed in the asymptomatic phase of pleural empyema. All patients were assessed in the phase of full capacity without any features of active bronchopleural fistula, as verified by bronchofiberoscopy.

### 2.2. Measurements

Because of the long observation time, scintigraphy was performed using one of three different gamma cameras: MB 9200-Gamma Muvex gamma camera with NMS software and the AntScan-Mediso and Nucline AP-Mediso gamma cameras with InterViewXP software. Scan results from all devices were standardized for further analysis.

Lung perfusion scintigraphy was performed after intravenous administration of human macroalbumin (Macroalbumon) labeled with Technetium-99mTc at a dose of 130–300 MBq. Gamma camera readings were obtained in the planned positions 10 min after radioisotope administration. The patient was sitting during the perfusion scan.

Ventilation scintigraphy was performed 48–72 h after measuring perfusion. In a sitting position, the patient breathed in a closed system and inhaled an aerosol of (Solco Venticoll) albumin microspheres and DTPA (PoltechDTPA 13.25 mg) labeled with Technetium-99mTc at a dose of 600–1000 MBq for 10 min. Gamma camera readings were obtained in the planned positions immediately after the inhalation stage as described in the perfusion study. Because of the deposition of some of the radioisotope on the surface of the inhalation system tubes, the initial isotope dose the ventilation study exceeded in the isotope dose in the perfusion study by >2–3 times.

### 2.3. Data Analysis

The obtained results are presented in typical projections: anterior, posterior, lateral, and diagonal. The calibration of the isotopic activity areas of the lung silhouette is determined in the IT system by the physician performing the examination, and the program automatically determines the activity levels in individual fields and in the entire lung. Moreover, three-dimensional imaging was possible using single-photon emission computed tomography (SPECT) supplemented with computed tomography (CT) and magnetic resonance [5]. We used a planar system for average radioactivity distribution recording with the lung silhouette divided into three equal fields (upper, middle, and lower) in the postero-anterior projection, corresponding to the percent of total lung perfusion and ventilation in individual lung fields. The results for perfusion and ventilation were presented as separate data sets. V/Q was determined for both the entire lung and individual lung fields.

MS Excel 2007 (Microsoft Corp., Redmond, WA, USA) and STATISTICA 13 (StatSoft Inc., Tulsa, OK, USA) were used for data preparation and statistical analysis. The normality of the data was examined using the Shapiro–Wilk test. In further comparisons, due to the non-normal distribution of the analyzed data sets, the Mann–Whitney U test was used. Correlations between examined features in the study group and subgroups were assessed using Spearman’s rank correlation coefficient. The level of significance was assumed to be *p* < 0.05.

## 3. Results

### 3.1. Perfusion

**Figure 3:**
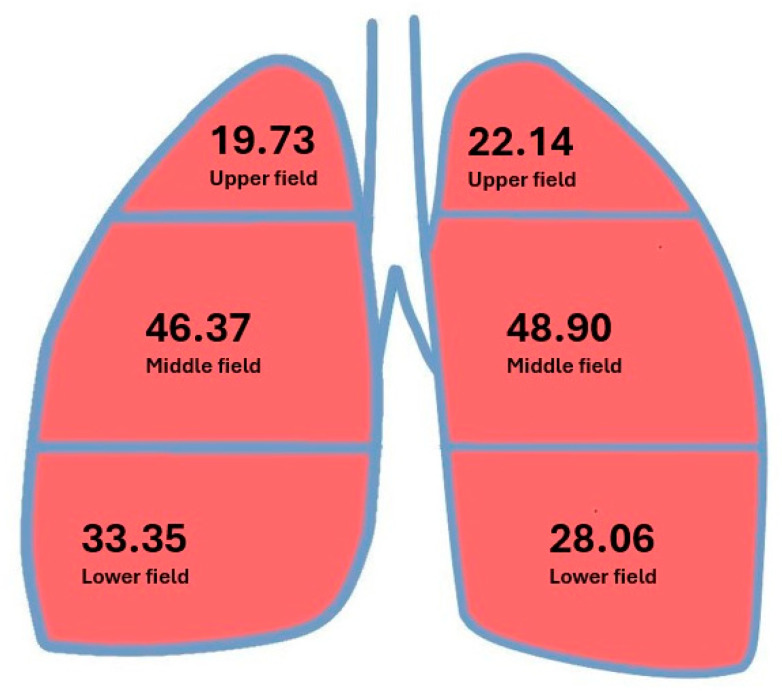
Mean flow values in individual lung fields.

Perfusion values are provided in Figure 3 and Table 2, Table 3 and Table 4.

The mean flow value in the upper field of the right lung did not exceed 20% of total lung perfusion, and flow in the combined lower and middle fields was almost 80%.

The mean flow value in the upper field of the left lung exceeded 20% of total lung perfusion, and perfusion in the combined middle and lower fields was <80% of total lung perfusion.

The mean flow value in the upper field regardless of side exceeded 20% of total lung perfusion, and perfusion in the combined middle and lower fields was <80% of total lung perfusion.

### 3.2. Ventilation

**Figure 4:**
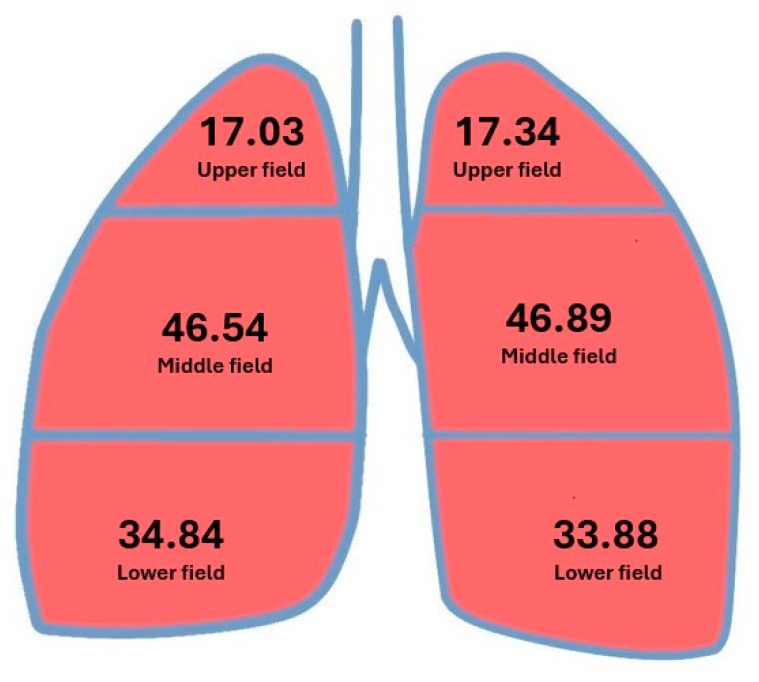
Mean ventilation values in individual lung fields.

Ventilation values in individual lung fields are presented in Figure 4 and Table 5.

The mean ventilation value in the upper field of the right lung did not exceed 18% of the total lung ventilation, and ventilation in the combined middle and lower fields exceeded 80% of the total lung ventilation. The mean ventilation value in the lower field exceeded the ventilation in the upper field by >2 times (34.84% vs. 17.03%).

The mean ventilation values in the upper field of the left lung did not exceed 18% of the total lung ventilation, and ventilation in the combined middle and lower fields slightly exceeded 80% of the total lung ventilation. The mean ventilation value in the lower field exceeded the ventilation in the upper field by almost 2 times (33.88% vs. 17.34%).

The mean ventilation values in the upper lung field did not exceed 18% of the total lung ventilation regardless of side. Ventilation in the combined middle and lower lung fields slightly exceeded 80% of the total lung ventilation, and ventilation in the lower field exceeded the ventilation in the upper field by nearly 2 times (34.28% vs. 17.21%).

### 3.3. Ventilation–Perfusion Ratio

**Figure 5:**
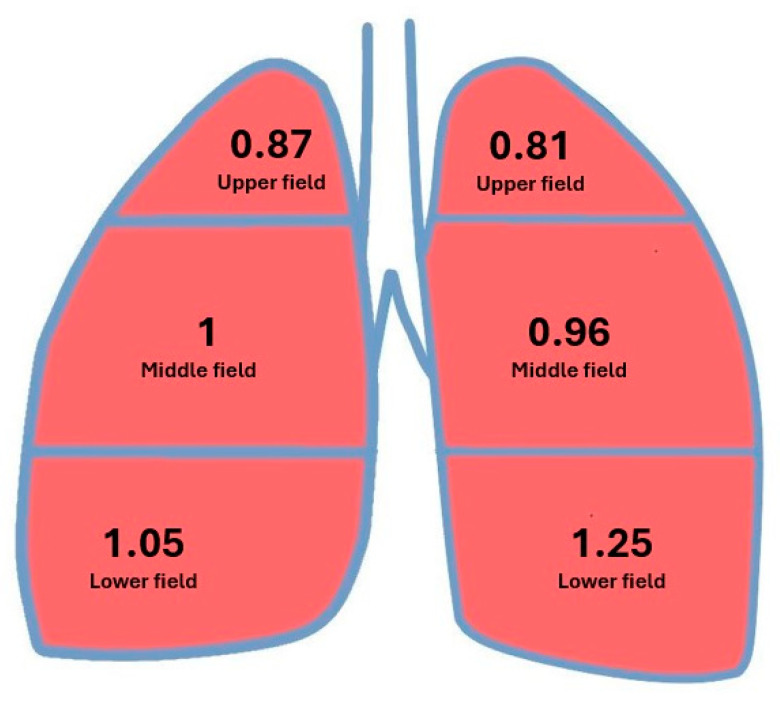
Mean ventilation–perfusion ratios in individual fields of the left and right lungs.

The V/Q in individual fields in the right and left lung and the ratio of the V/Q value in the lower field to the V/Q value in the upper field (lower field/upper field—L/U) regardless of the side examined are presented in Figure 5 and Table 6 and Table 7.

The mean value of the V/Q in the upper field of the right lung did not exceed 1.0, whereas the mean value of the V/Q in the middle field was 1, and the mean value of the V/Q in the lower field and the combined middle and lower fields exceeded 1.0.

The mean value of the V/Q in the upper and middle fields of the left lung did not exceed 1.0, whereas the mean value of the V/Q in both the lower field and the combined middle and lower fields exceeded 1.0.

The mean value of the V/Q in the upper and middle lung fields did not exceed 1.0, whereas the mean value of the V/Q in both the lower lung field and the combined middle and lower lung fields exceeded 1.0.

The mean value of the V/Q ratio in the upper field was 0.84, whereas the mean V/Q ratio in the lower field was 1.16. The mean value of the ratio of V/Q in the lower lung field to V/Q in the upper lung field (L/U) was 1.38. The minimal value of the L/U ratio was 0.5, whereas the maximal value was 2.83. The standard deviation was 0.64.

**Figure 6:**
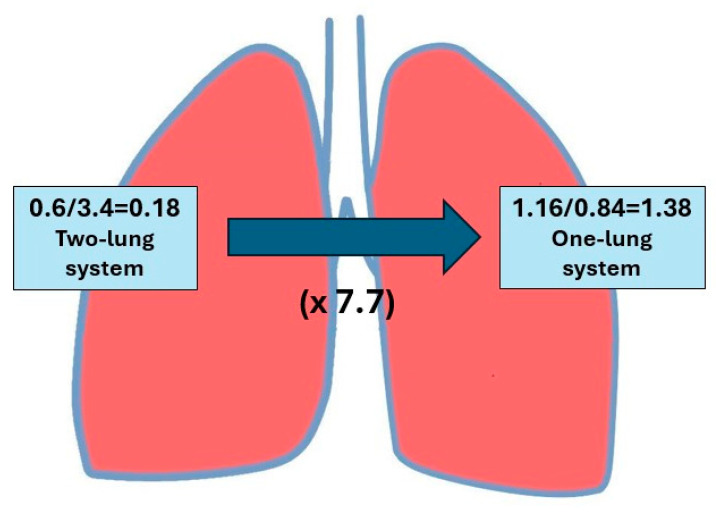
The ratio of the ventilation–perfusion ratio at the base of the lung to the ratio in the apex in the two lungs under physiological conditions (two-lung system) compared with the remaining lung after pneumonectomy (single-lung system).

The ratio of the mean V/Q value in the lower field (1.16) to the mean V/Q in the upper field (0.84) regardless of side was 1.38. Under physiological conditions in the two lungs before pneumonectomy (two-lung system), the ratio of V/Q at the base of the lung (0.6) to V/Q in the apex (3.4) was 0.18, indicating a 7.7-fold increase (1.39/0.18 = 7.7) in the remaining lung (single-lung system) compared to physiological norms (Figure 6). The increase was greater on the left side (1.54/0.18 = 8.5) than on the right side (1.20/0.18 = 6.7). Perfusion, ventilation, and V/Q by operated side are presented in Table 7, Table 8, Table 9, Table 10 and Table 11.

The mean perfusion value in the lower field of the right lung was significantly higher than the perfusion in the corresponding field of the left lung (*p* = 0.001). No significant difference was found between the right and left sides for the perfusion in other lung fields.

The mean ventilation values in lung fields were not significantly different between the right and left sides.

Comparative analysis of the mean V/Q in individual lung fields between the right and left sides showed that, in the upper pulmonary fields, V/Q values were <1.0 and in the lower pulmonary fields, they were >1.0. The mean value of V/Q in the lower field of the left lung was significantly higher than the value in the lower field of the right lung (1.25 vs. 1.05, *p* = 0.034). In other fields, comparisons between the right and left lungs did not reveal significant differences.

The difference in L/U between the right and left lungs approached significance (*p* = 0.058).

The mean V/Q (Table 11) exceeded the literature norms (0.8–0.9, mean 0.85).

### 3.4. Results Depending on Time from Pneumonectomy and Sex: Correlations

The results of ventilation, perfusion, and V/Q ratio depending on time from pneumonectomy are presented in Table 12.

The results of perfusion and ventilation and V/Q ratio depending on sex are presented in Table 13, Table 14 and Table 15.

No significant differences in perfusion, ventilation, or V/Q were found depending on sex and time from pneumonectomy to scintigraphy.

The nature of correlations between select flow parameters, ventilation parameters, and other features was determined regardless of which side was examined. A positive correlation was found between upper-field ventilation and the upper-field V/Q (0.73, *p* < 0.0001) and between ventilation and flow in the combined middle and lower fields (0.45, *p* = 0.01). In addition, a number of negative correlations were found. For example, we identified a negative correlation between ventilation in the upper field and the V/Q of the combined middle and lower fields (−0.59, *p* = 0.0004) and between ventilation in the combined middle and lower fields and the V/Q of the upper field (−0.61, *p* = 0.0002). Furthermore, a negative correlation was found between the V/Q in the upper field and the V/Q in the combined middle and lower fields (0.91, *p* < 0.0001), as well as between the V/Q in the upper field and the V/Q in the lower field (−0.68, *p* < 0.0001). A negative correlation was also found between the ventilation in the upper field and the ventilation in the combined middle and lower fields (−0.92, *p* < 0.0001) and between the ventilation values in the upper and lower fields (−0.42, *p* = 0.018). In the lower field, a strong negative correlation was found between the V/Q and the flow in this field (−0.71, *p* < 0.0001). However, a positive correlation was found between the V/Q and the ventilation in the lower field (0.4, *p* = 0.027). Perfusion in the upper field had a negative correlation with the V/Q in the upper field (−0.42, *p* = 0.02) and with perfusion in the lower field (−0.66, *p* < 0.0001) and perfusion in the combined middle and lower fields (−0.96, *p* < 0.0001). Flow in the lower field had a positive correlation with the V/Q in the upper field (0.46, *p* = 0.008). No significant correlations were found between perfusion and ventilation in the upper field (0.23, *p* = 0.2). No significant correlations were found between perfusion, ventilation, and V/Q depending on time from pneumonectomy.

## 4. Discussion

The popularity of pneumonectomy as a treatment for lung cancer has significantly declined in favor of other less extensive lung resections, especially those utilizing minimally invasive techniques [10,11,12,13,14]. According to the German Thorax Registry Study, the in-hospital mortality after pneumonectomy is 7% in general but 25% among cases with pulmonary complications [15]. Therefore, the number of novel reports on functional assessments of the single remaining lung is rather low [16,17]. Over 80 years of experience in this group of patients has allowed the long-term consequences of pneumonectomy to be described. These consequences include postural deformities associated with scoliosis and autothoracoplasty on the operated side, compensatory emphysema of the remaining lung with a shift of the mediastinum to the operated side, and anatomical changes (e.g., augmentation of the right ventricle and right atrium, dilatation of the pulmonary artery) associated with the development of pulmonary hypertension and lower tolerance of physical activity [18,19,20,21,22,23,24,25,26]. On the other hand, many patients with one lung demonstrate cardiorespiratory performance allowing for regular physical activity, even playing sports, which justifies continuation of the functional assessment [25,27]. Available studies have focused on the analysis of ventilation function by spirometry, the analysis of gas diffusion, blood oxygenation levels, and exercise tolerance, and echocardiographic assessment of the heart and pulmonary artery [17,27,28,29]. Analysis of the ventilation and perfusion of the lungs is currently possible using scintigraphic techniques [3,4,5,6,17,30]. Combining ventilation and perfusion enables determination of the V/Q [3,9], which allows each lung to be analyzed in terms of selected areas of activity and, thus, the efficiency of the two most important respiratory functions, as well as the respiratory function of the lungs as a whole. Combined isotope studies have been used to predict postoperative lung function, which is useful in planning the range of lung resection [29,31,32,33]. Moneke et al. demonstrated that perfusion SPECT/CT can be used to predict lung function after resection with efficacy similar to perfusion scintigraphy [34]. Ventilation SPECT/CT is another diagnostic modality with similar efficacy and can be used to predict lung function after resection for lung cancer. Jeong and Lee reported that postoperative forced expiratory volume in 1 s (FEV_1_) and DLCO values predicted by ventilation SPECT/CT strongly correlated with those predicted by perfusion SPECT/CT (correlation coefficient r = 0.939 for postoperative FEV_1_%, *p* < 0.001; r = 0.938 for postoperative DLCO%, *p* < 0.001) [35].

Postoperative isotope assessment of lung function has been the subject of numerous studies at our center. Two such studies included a group of patients who underwent pneumonectomy. In both studies, perfusion scintigraphy was used to assess the pulmonary blood flow in the remaining lung [36,37]. The current study is a continuation of previous research on single-lung function, but we utilized a ventilation scan in addition to the perfusion scan.

The perfusion results were consistent with previous reports, which showed that flow increases in each lung field after pneumonectomy, with possible further variation in flow depending on the side of the examination and the perfusion of individual lung regions. According to the literature, the flow in the combined lower and middle pulmonary fields (mean 78.16%) is higher than the flow in the upper pulmonary field (mean 21.1%); however, the flow in the upper field is more than twice the physiological flow in this field (<10%) for the two-lung system, indicating increased utilization of the flow reserve [2,36,37,38]. Increased flow in the upper field of the remaining lung is one of the initial mechanisms counteracting the development of pulmonary hypertension after pneumonectomy and, during longer follow-up, flow in the upper field reflects the use of the flow reserve in the remaining lung [16,36,37]. We also demonstrated differences in pulmonary flow in individual lung fields depending on the operated side. The mean flow in the upper field of the right lung was lower than the mean flow in the upper field of the left lung (19.73 vs. 22.14, *p* = 0.06), and the mean flow in the combined middle and lower fields of the right lung was higher than the mean flow in the combined middle and lower fields of the left lung (79.72 vs. 76.95, *p* = 0.071). Both observations did not reach significance, but they may be real and related to the physiological difference in the size of the right (50–55% of pulmonary perfusion) and left lungs (45–50% of pulmonary perfusion) [39]. Significant differences were found in the flow in the lower field. The mean perfusion in the lower field of the right lung was significantly higher than the mean perfusion in the lower field of the left lung (33.35 vs. 28.05, *p* = 0.001; Table 13), which can also be explained by the difference in volume between the right and left lungs and the effect of gravity on pulmonary blood flow [39]. These observations were also reflected in the analysis of the correlation of flow factors, as the dependence of the flow in the upper field in relation to the flow in the lower field (−0.66, *p* < 0.0001) and in the combined middle and lower fields (−0.96, *p* < 0.001) showed negative and significant correlations regardless of side. Moreover there was also a negative correlation between flow in the upper field and flow in the combined middle lower and middle fields on the right (−0.97, *p* < 0.001) and left sides (−0.9, *p* < 0.001).

Unlike the results for perfusion, ventilation was very similar for the right and left lungs. The mean ventilation value in the upper pulmonary field ranged from 17% to 18%, and in the combined middle and lower pulmonary fields, it ranged from 80% to 81.5%. Interestingly, this value was almost identical in the upper and middle fields of the right and left lungs, and the ventilation values in individual fields of the right and left lungs did not differ significantly (Table 5 and Table 8). Limiting ventilation to a single lung was associated with a greater ratio of ventilation in the lower fields to ventilation in the upper fields compared to a two-lung system (~2 vs. 1.5). According to Konturek, basal ventilation can be up to 3-times greater than apex ventilation, though such a comparison concerns lung areas smaller than a lobe [9]. Higher ventilation and perfusion in the combined middle and lower fields compared with the upper field mimic the conditions previously reported in a two-lung system [17,40]. The introduction of aerosol scintigraphy revealed the phenomenon of so-called hot spots on the image of the examined area. This phenomenon is caused by an accumulation of radiopharmaceuticals in areas of flow obstruction in the bronchial tree. This phenomenon did not affect the results obtained in our study group because the condition of the bronchial tree was examined by bronchofiberoscopy.

A number of compensatory processes occur after pneumonectomy [41,42]. For example, so-called compensatory emphysema develops due to excessive expansion of the remaining lung parenchyma, compensating for the loss of lung volume [41,43,44,45,46,47,48,49]. According to Mergoni and Rossi, compensatory emphysema is characterized by ventilation of the remaining lung at a lower intrathoracic pressure and lesser changes in gas exchange compared to emphysema in the course of, for example, chronic obstructive pulmonary disease, in which an increased lung volume is accompanied by normal intrathoracic pressure and impaired gas diffusion. This situation positively affects the efficiency of ventilation in patients with one lung [50].

Interestingly, the postoperative increase in lung volume depends on the type of resection. Shibazaki et al. reported that expansion of the middle lobe was greater after right lower lobectomy than after right upper lobectomy [51]. In another study, among patients observed for 3 to 20 years after pneumonectomy, the vast majority had lung volumes greater than expected based on the amount of parenchyma removed. In these patients, total lung capacity and forced vital capacity (FVC) were within the normal range and exhibited compensatory growth [49]. Shibazaki reported a positive correlation between the residual lung expansion ratio and FEV1 ratio [52]. In a study by Topaloglu et al., a cohort with a postoperative residual lung volume ratio of at least 1.2 had a higher postoperative FVC [45].

Radiological features of compensatory emphysema typically appear 4–5 years after pneumonectomy. Compensatory emphysema appeared in our study group, but we did not find significant differences in flow, ventilation, or the V/Q in the upper, lower, and combined lower and middle pulmonary fields according to time from pneumonectomy.

Maintenance of normal respiratory function requires adequate ventilation combined with optimal pulmonary flow. Impaired flow in the presence of normal, or even increased, ventilation creates a state of so-called dead space, whereas insufficient ventilation but maintained flow leads to the development of a so-called pulmonary shunt with ventilation–perfusion mismatch [53,54].

The normal mean V/Q for the entire lungs is 0.8–0.9. The V/Q differs between the apical and basal parts of the lung due to individual lung areas being located above or below the level of the heart. Both blood flow and ventilation increase from the apical to the basal segments of the lung, but the increase in perfusion is greater, mainly due to gravity and the hydrostatic pressure of blood. The perfusion pressure at the base of the lung is equal to the sum of the mean pulmonary artery pressure and the difference between the hydrostatic pressure at the level of the heart and the value of this pressure at the base of the lung [9]. The influence of hydrostatic pressure is reduced in the apical segments of the lung, which can result in low flow pressure. Moreover, hydrostatic pressure sometimes reaches values lower than the pressure in the alveoli, leading to vascular compression and flow disruption, constituting the area of the so-called lung perfusion reserve [9].

Ventilation values in the lungs depend on their compliance (so-called lung distensibility index). The lung base has a better ability to change the volume and intensity of ventilation [55]. The lower lung fields are ventilated approximately 1.5-times more intensively than the upper lung fields, with a greater change in their volume. A 9-fold increase in perfusion in the basal regions of the lungs compared to the apical regions results in an increased V/Q in the apical fields (3.4) and a decreased V/Q at the lung base (0.6) [8,9]. A V/Q > 3 in the upper pulmonary field indicates a large, preserved perfusion reserve (i.e., <10% of the total perfusion of a given lung), which occurs most often in young individuals without any chronic diseases, who have optimal respiratory performance. Patients in the present study were in the 5th to 8th decade of life, with possible changes in pulmonary perfusion and ventilation, but they still met the strict eligibility criteria for pneumonectomy.

In healthy individuals with a two-lung system, effective pulmonary flow is >94% of the total perfusion, and effective alveolar ventilation is >70% of total ventilation [53]. In the present study, a significant increase in both perfusion and ventilation was demonstrated in the remaining lung. Mean perfusion values were 99.44% for the right side and 99.09% for the left side. Mean ventilation values were 98.37% for the right side and 98.10% for the left side. The obtained results demonstrate the degree of intensification of respiratory processes in the single-lung system. The mean V/Q regardless of operated side was 0.98, indicating a rather proportional increase in both ratio components in the assessment of both the right and left lungs, which was consistent with the results of the study published by Martin [40]. However, this exceeded the literature norms (0.8–0.9, mean 0.85), but none of the mean V/Q values (right, left, either) exceeded 1.0.

Further analysis revealed both similarities and differences in the mean V/Q values for individual lung fields. Differences concerned the values in the upper pulmonary fields compared to the values in the middle, combined middle and lower, and lower pulmonary fields, as well as between the lower fields of both lungs. Similarities were observed for parallel fields assessed in both lungs. The V/Q in the upper fields of both lungs ranged from 0.8 to 0.9, whereas in the middle lung fields it was approximately 1 (0.96–1). The mean V/Q values in the lower fields, but also in the combined middle and lower fields, exceeded 1. This is in contrast to the distribution of V/Q values described in the literature. The V/Q value in the upper field was reduced 4-fold (0.84 vs. 3.4), whereas the V/Q value in the lower field increased nearly 2-fold (1.16 vs. 0.6), which reflects the increase in the V/Q from the apical to the basal segments of the lung.

The reason for the described changes is an increase (up to >2-fold) in the upper field flow and increase (nearly 2-fold) in the ventilation in the lower field of the examined lung. Significant differences between the right and left side were demonstrated for the V/Q in the lower field (1.05 vs. 1.25, *p* = 0.034). The lower V/Q in the lower field on the right side indicates a relative increase in perfusion, which is reflected by the higher flow in the lower field on the right side compared to the left side (33.35 vs. 28.05, *p* = 0.001). Analysis of the association between perfusion, ventilation, and the V/Q revealed a negative correlation of V/Q in the upper field with perfusion in the upper field (−0.42, *p* = 0.02), as well as a strong, positive correlation of V/Q in the upper field with ventilation in the upper field (0.73, *p* < 0.0001). Similarly, a strong negative correlation of the V/Q in the lower field with lower field perfusion (−0.71, *p* < 0.0001) was demonstrated as well as a positive correlation of the V/Q in the lower field with lower field ventilation (0.40, *p* = 0.027).

Under physiological conditions in the two-lung system, the ratio of the V/Q in the basal regions (0.6) to the V/Q in the apical regions (3.4) is 0.18. The mean value of the ratio of V/Q in the lower field to the V/Q in the upper field in the study group was 1.38, indicating a 7.7-fold increase in the one-lung system compared to physiological norms. The increase was greater on the left side (1.54/0.18 = 8.5) than on the right side (1.20/0.18 = 6.7) (Figure 6, Table 9).

The obtained results describe the principle of mutual dependence between processes occurring in the assessed lung areas. Processes occurring in the upper lung field and in the rest of the lung are in opposing relationships, which causes an increase in perfusion and ventilation in the upper lung field to be accompanied by a decrease in perfusion and ventilation in the remaining lung area. According to this principle, a strong negative correlation was demonstrated between the V/Q values in the upper lung field and the combined middle and lower lung fields (−0.91, *p* < 0.0001), as well as between the upper and lower lung fields (−0.68, *p* < 0.0001). Negative correlations concerned the relationships with the V/Q and the mutual relationships of perfusion and ventilation between the individual lung fields. At the same time, no correlations were found between perfusion and ventilation within the upper field (0.23, *p* = 0.2), though such a relationship was found within the combined middle and lower fields (0.45, *p* = 0.01). Furthermore, no correlations were found for the V/Q depending on time from pneumonectomy.

The results of the present study provide insight into the processes occurring in the remaining lung after pneumonectomy. A sudden increase in blood flow in the remaining lung alters the perfusion conditions of the lung and forces the use of flow reserves, which is especially visible in the upper field. The differences between the left and right lung in this regard should be investigated further. Moreover, greater perfusion in the lower field of the right lung compared to the lower field of the left lung could be associated with anatomical differences between the right and left lung.

Changes in ventilation develop differently. Here, increased lower lung field ventilation occurred without significant differences between the right and left lung, which together with changes in perfusion influences the characteristics of the V/Q. The ratio did not exceed physiological limits within the upper lung fields and increased towards the basal pulmonary regions. Significant differences in flow in the lower field between the right and left side contributed to significantly larger values of the V/Q on the left side. Changes in the V/Q in the upper and lower fields indicate a blurring of the differences between these fields and an intensification of ventilation and perfusion in the single lung after pneumonectomy. In further follow-up, the practical implications of the obtained results may be significant in cases of infiltrative diseases of a single lung, with the development of V/Q mismatch, or in predictive assessment of qualification for procedures known as resections greater than pneumonectomy [56]. Moreover, the understanding of the changes in ventilation and perfusion measured by means of scintigraphy after pneumonectomy paves the way for further research on the use of ventilation/perfusion scintigraphy in monitoring patients after pneumonectomy. V/Q scan, if possible in combination with echocardiography, could be used in further research to assess the development of pulmonary hypertension and its pharmacological control after pneumonectomy.

The main limitation of this evaluation is the small size of the study group and the heterogeneity of the interval between pneumonectomy and scintigraphy. Because of decreasing number of pneumonectomies, this type of analysis may require multicenter studies in the future.

## 5. Conclusions

In a group of patients who underwent pneumonectomy, we found increased perfusion in the upper pulmonary field and increased ventilation in the lower pulmonary field compared to the expected norms for the physiological system with two lungs. We also demonstrated a reversal of the V/Q between the upper and lower fields, reflecting maximum use of the reserves for ventilation and perfusion in the remaining lung. Comparing the right and left lung revealed greater perfusion in the lower field of the right lung with a larger V/Q value in the lower field of the left lung but with no differences in ventilation between the right and left sides. The results of this study pave the way for further research on larger groups of patients.

## Figures and Tables

**Table 1:** Characteristics of the study group (N = 30).

Characteristic	Value
Male, n	25
Female, n	5
Age, range, years	47–74
Average age, years	61.6
Operated side—right, n	17
Operated side—left, n	13
Operated for oncological reasons, n	28
Lung cancer (squamous-cell carcinoma)	18
Lung cancer (adenocarcinoma)	8
Lung cancer (adenosquamous carcinoma)	1
Carcinoid with infiltration	1
Stage IB	1
Stage IIA	2
Stage IIB	16
Stage IIIA	6
Stage IIIB	3
Operated on for non-oncological reasons, n	2
Non-specific lung abscess	1
Gunshot wound of the lung	1
Completed treatment for pleural empyema, n (%)	25 (83%)
Duration of empyema treatment, range/mean, months	1.5–541/43.8
Duration of empyema treatment, range/mean value for Szczecin center, months	1.5–173.6/24.4
Time from pneumonectomy to scintigraphy, range, days	141–16,469
Average/median time from pneumonectomy to scintigraphy, days	1893/1100
Height, range/mean, cm	146–196/170
Weight, range/mean, kg	36–105/75
BMI, range/mean, kg/m^2^	16.8–35.8/25.9

**Table 2:** Perfusion in individual lung fields of the right lung.

Lung Field	Mean Perfusion	Minimal Perfusion	Maximal Perfusion	Standard Deviation
Upper field	19.73	14.86	27.68	3.8
Middle field	46.37	40	54.72	4.26
Lower field	33.35	26.86	38.3	3.24
Combined lower and middle fields	79.72	71.8	85.02	4.24

**Table 3:** Perfusion in individual fields of the left lung.

Lung Field	Mean Perfusion	Minimal Perfusion	Maximal Perfusion	Standard Deviation
Upper field	22.14	13.22	28.5	4.17
Middle field	48.90	40.6	60.07	4.66
Lower field	28.06	18.76	42.2	5.58
Combined lower and middle fields	76.96	68.9	86.69	4.59

**Table 4:** Perfusion in individual lung fields regardless of side.

Lung Field	Mean Perfusion	Minimal Perfusion	Maximal Perfusion	Standard Deviation
Upper field	21.1	13.22	28.5	4.13
Middle field	47.80	40	60.07	4.6
Lower field	30.35	18.76	42.2	5.35
Lower and middle field	78.16	68.9	86.69	4.59

**Table 5:** Ventilation in individual fields of the right lung, left lung, and entire study group.

Examined Side	Lung Field	Mean Ventilation	Minimal Ventilation	Maximal Ventilation	Standard Deviation
Right	Upper field	17.03	11.63	24.84	4.21
	Middle field	46.54	34.1	56.83	6.27
	Lower field	34.8	26.86	40.1	3.33
	Combined middle and lower fields	81.34	71.4	88.32	5.83
Left	Upper field	17.34	10.09	23.54	4.21
	Middle field	46.89	35.3	57.57	6.16
	Lower field	33.88	26.68	43.21	4.88
	Combined middle and lower fields	80.77	73.9	89.85	5.29
Entire study group	Upper field	17.21	10.09	24.84	4.14
	Middle field	46.73	34.1	57.57	6.11
	Lower field	34.28	26.68	43.21	4.24
	Combined middle and lower fields	81.01	71.4	89.85	5.44

**Table 6:** Ventilation–perfusion ratios (V/Q) in individual fields of the right lung, left lung, and entire study group.

Examined Side	Lung Field	Mean V/Q	Minimal V/Q	Maximal V/Q	Standard Deviation
Right	Upper field	0.87	0.54	1.17	0.19
	Middle field	1.0	0.84	1.09	0.06
	Lower field	1.05	0.89	1.28	0.11
	Combined middle and lower fields	1.02	0.92	1.13	0.06
Left	Upper field	0.81	0.49	1.78	0.30
	Middle field	0.96	0.76	1.15	0.11
	Lower field	1.25	0.85	1.97	0.28
	Combined lower and middle fields	1.05	0.88	1.17	0.07
Entire study group	Upper field	0.84	0.49	1.78	0.26
	Middle field	0.98	0.76	1.15	0.1
	Lower field	1.16	0.85	1.97	0.24
	Combined middle and lower fields	1.04	0.88	1.17	0.07

**Table 7:** Comparison of perfusion in individual pulmonary fields and combined middle and lower fields.

Lung Field	Examined Side	Mean Perfusion	Minimal Perfusion	Maximal Perfusion	Standard Deviation	*p* Value
Upper field	Right	19.72	14.86	27.68	3.80	0.06
	Left	22.14	13.22	28.5	4.16	
Middle field	Right	46.36	40	54.72	4.2	0.15
	Left	48.90	40.6	60.07	4.6	
Lower field	Right	33.35	26.86	38.3	3.2	0.001
	Left	28.05	18.76	42.2	5.5	
Combined middle and lower fields	Right	79.72	71.8	85.02	4.2	0.071
	Left	76.95	68.9	86.69	4.5	

**Table 8:** Comparison of ventilation in individual pulmonary fields and in the combined middle and lower fields.

Lung Field	Examined Side	Mean Ventilation	Minimal Ventilation	Maximal Ventilation	Standard Deviation	*p* Value
Upper field	Right	17.03	11.63	24.84	4.2	0.83
	Left	17.34	10.09	23.54	4.2	
Middle field	Right	46.54	34.1	56.83	6.2	0.80
	Left	46.88	35.3	57.57	6.1	
Lower field	Right	34.79	26.86	40.1	3.3	0.62
	Left	33.87	26.68	43.21	4.8	
Combined middle and lower fields	Right	81.34	75.11	88.32	0.06	0.77
	Left	80.76	73.90	89.85	0.07	

**Table 9:** Comparison of the ventilation–perfusion ratio (V/Q) in individual pulmonary fields and in the combined middle and lower fields.

Lung Field	Examined Side	Mean V/Q	Minimal V/Q	Maximal V/Q	Standard Deviation	*p* Value
Upper field	Right lung	0.87	0.54	1.16	0.18	0.24
	Left lung	0.81	0.49	1.78	0.30	
Middle field	Right	1.0	0.84	1.09	0.06	0.22
	Left	0.96	0.76	1.15	0.11	
Lower field	Right	1.05	0.88	1.28	0.10	0.034
	Left	1.25	0.85	1.96	0.27	
Combined middle and lower fields	Right	1.02	0.91	1.12	0.06	0.14
	Left	1.05	0.87	1.17	0.07	

**Table 10:** Comparison of the ventilation–perfusion ratio (V/Q) in the lower pulmonary field to V/Q in the upper pulmonary field (L/U).

Examined Side	Mean V/Q, Upper Field	Mean V/Q, Lower Field	L/U	Standard Deviation	*p* Value
Right	0.87	1.05	1.20	0.44	0.058
Left	0.81	1.25	1.54	0.71	

**Table 11:** Comparison of perfusion (V) and ventilation (Q) in the entire lung.

Examined Side	Total Perfusion	Total Ventilation	V/Q
Right	99.44	98.37	0.98
Left	99.09	98.10	0.98
Either	99.25	98.22	0.98

**Table 12:** Perfusion ventilation and V/Q ratio depending on time from pneumonectomy (<5 years versus > 5 years).

Lung Field	Time from Pneumonectomy	Number of Patients	Perfusion	Ventilation	V/Q Ratio
Upper field	<5 years	21	20.59	15.59	0.83
	>5 years	9	22.27	18.65	0.84
Lower field	<5 years	21	31.18	34.41	1.13
	>5 years	9	28.42	33.97	1.23
Combined middle and lower fields	<5 years	21	78.61	81.57	1.04
	>5 years	9	77.07	79.72	1.04

**Table 13:** Perfusion in individual lung fields depending on sex.

Lung Field	Female	Male	*p* Value
Upper field	21.83	20.94	0.55
Middle field	47.43	47.87	0.58
Lower field	29.64	30.49	0.95
Combined middle and lower fields	77.07	78.37	0.51

**Table 14:** Ventilation of individual lung fields depending on sex.

Lung Field	Female	Male	*p* Value
Upper field	15.66	17.51	0.51
Middle field	45.68	46.94	0.48
Lower field	35.59	34.01	0.27
Combined middle and lower fields	81.27	80.96	0.91

**Table 15:** V/Q ratio of individual lung fields depending on sex.

Lung Field	Female	Male	*p* Value
Upper field	0.74	0.85	0.38
Lower field	1.28	1.13	0.55
Combined middle and lower fields	1.05	1.03	0.44

## Data Availability

Data available on request due to ethical reasons. Patient’s records are kept in the archives of the Department of Thoracic Surgery and Transplantation, Pomeranian Medical University, Szczecin, Poland.

## Abbreviations

The following abbreviations are used in this manuscript:

BMI	Body mass index
SPECT	single-photon emission computed tomography
CT	Computed tomography
MR	Magnetic resonance
FEV1	Forced expiratory volume in 1 s
FVC	Forced vital capacity

## References

[1] Kodgule R., Vanjare N., Rasam S., Bhosale S., Gupta Y., Salvi S. (2014). Correlation of Spirometry with Impulse Oscillometry, Body Plethysmography and DLCO. Eur. Respir. J..

[2] Wójcik J., Sedlaczek A., Grodzki T., Urbański S. (1998). Pulmonary Flow in Patients Subjected to Lung Decortication Due to Chronic Pleural Empyema. Pol. J. Surg..

[3] Krawczyk-Sulisz I., Andrzej M.S., Grodzki T. (1998). Radioisotope Evaluation of Ventilation and Perfusion after Late Lung Decortication Due to a Chronic Pleural Empyema. Pol. Przegląd Chir..

[4] Dupuis J., Harel F., Nguyen Q.T. (2014). Molecular Imaging of the Pulmonary Circulation in Health and Disease. Clin. Transl. Imaging.

[5] Chun K.K. (2015). Nuclear Medicine and PET/CT Cases.

[6] Amis T.C., Crawford A.B., Davison A., Engel L.A. (1990). Distribution of Inhaled 99mtechnetium Labelled Ultrafine Carbon Particle Aerosol (Technegas) in Human Lungs. Eur. Respir. J..

[7] Mortensen J., Berg R.M.G. (2019). Lung Scintigraphy in COPD. Semin. Nucl. Med..

[8] Dziuk E. (1978). Radioizotopowe Metody Badania Układu Oddechowego.

[9] Brzozowski T. (2019). Konturek Human Physiology.

[10] Yang C.-F.J., D’Amico T.A. (2012). Thoracoscopic Segmentectomy for Lung Cancer. Ann. Thorac. Surg..

[11] Jones C.D., Cummings I.G., Shipolini A.R., McCormack D.J. (2013). Does Surgery Improve Prognosis in Patients with Small-Cell Lung Carcinoma?. Interact. Cardiovasc. Thorac. Surg..

[12] Yuequan J., Zhi Z., Chenmin X. (2012). Surgical Resection for Small Cell Lung Cancer: Pneumonectomy versus Lobectomy. ISRN Surg..

[13] Eguchi T., Kumeda H., Miura K., Hamanaka K., Shimizu K. (2024). Saving Lives in Thoracic Surgery: Balancing Oncological Radicality and Functional Preservation, Transitioning from Standard Pneumonectomy to Targeted Sublobar Resection. Cancers.

[14] Kim N., Priefer R. (2021). Drug Regimen for Patients after a Pneumonectomy. J. Respir..

[15] Semmelmann A., Baar W., Fellmann N., Moneke I., Loop T. (2023). Working Group of the German Thorax Registry. The Impact of Postoperative Pulmonary Complications on Perioperative Outcomes in Patients Undergoing Pneumonectomy: A Multicenter Retrospective Cohort Study of the German Thorax Registry. J. Clin. Med..

[16] Anthonisen N.R., Bass H., Heckscher T. (1968). 133Xe Studies of Patients after Pneumonectomy. Scand. J. Respir. Dis..

[17] Hall D.R. (1974). Regional Lung Function after Pneumonectomy. Thorax.

[18] Stępkowski M., Wojtyś M.E., Wójcik N., Safranow K., Pieróg J., Kordykiewicz D., Szulc J., Sulikowski T., Jarosz K., Grodzki T. (2025). Evaluation of Pulmonary Blood Flow, Right Atrium, Right Ventricle, and Pulmonary Artery in Patients After Pneumonectomy. J. Clin. Med..

[19] Saleh K., Khan N., Dougherty K., Bodi G., Michalickova M., Mohammed S., Kerenidi T., Sadik Z., Mallat J., Farha S. (2023). The First Pulmonary Hypertension Registry in the United Arab Emirates (UAEPH): Clinical Characteristics, Hemodynamic Parameters with Focus on Treatment and Outcomes for Patients with Group 1-PH. J. Clin. Med..

[20] Topyła-Putowska W., Tomaszewski M., Wysokiński A., Tomaszewski A. (2021). Echocardiography in Pulmonary Arterial Hypertension: Comprehensive Evaluation and Technical Considerations. J. Clin. Med..

[21] Hsia C.C., Carlin J.I., Cassidy S.S., Ramanathan M., Johnson R.L. (1990). Hemodynamic Changes after Pneumonectomy in the Exercising Foxhound. J. Appl. Physiol..

[22] Foroulis C.N., Kotoulas C.S., Kakouros S., Evangelatos G., Chassapis C., Konstantinou M., Lioulias A.G. (2004). Study on the Late Effect of Pneumonectomy on Right Heart Pressures Using Doppler Echocardiography. Eur. J. Cardiothorac Surg..

[23] Potaris K., Athanasiou A., Konstantinou M., Zaglavira P., Theodoridis D., Syrigos K.N. (2014). Pulmonary Hypertension after Pneumonectomy for Lung Cancer. Asian Cardiovasc. Thorac. Ann..

[24] El-Hamamsy I., Stevens L.-M., Perrault L.P., Carrier M. (2003). Right Pneumonectomy and Thoracoplasty Followed by Coronary Artery Bypass Grafting and Mitral Valve Replacement. J. Thorac. Cardiovasc. Surg..

[25] Soll C., Hahnloser D., Frauenfelder T., Russi E.W., Weder W., Kestenholz P.B. (2009). The Postpneumonectomy Syndrome: Clinical Presentation and Treatment. Eur. J. Cardiothorac Surg..

[26] Kowalewski J., Brocki M., Dryjański T., Kaproń K., Barcikowski S. (1999). Right Ventricular Morphology and Function after Pulmonary Resection. Eur. J. Cardiothorac Surg..

[27] Edvardsen E., Skjønsberg O.H., Holme I., Nordsletten L., Borchsenius F., Anderssen S.A. (2015). High-Intensity Training Following Lung Cancer Surgery: A Randomised Controlled Trial. Thorax.

[28] Riquet M., Mordant P., Pricopi C., Legras A., Foucault C., Dujon A., Arame A., Le Pimpec-Barthes F. (2014). A Review of 250 Ten-Year Survivors after Pneumonectomy for Non-Small-Cell Lung Cancer. Eur. J. Cardiothorac Surg..

[29] Nugent A.M., Steele I.C., Carragher A.M., McManus K., McGuigan J.A., Gibbons J.R., Riley M.S., Nicholls D.P. (1999). Effect of Thoracotomy and Lung Resection on Exercise Capacity in Patients with Lung Cancer. Thorax.

[30] Bajc M., Neilly J.B., Miniati M., Schuemichen C., Meignan M., Jonson B., EANM Committee (2009). EANM Guidelines for Ventilation/Perfusion Scintigraphy: Part 1. Pulmonary Imaging with Ventilation/Perfusion Single Photon Emission Tomography. Eur. J. Nucl. Med. Mol. Imaging.

[31] Win T., Tasker A.D., Groves A.M., White C., Ritchie A.J., Wells F.C., Laroche C.M. (2006). Ventilation-Perfusion Scintigraphy to Predict Postoperative Pulmonary Function in Lung Cancer Patients Undergoing Pneumonectomy. AJR Am. J. Roentgenol..

[32] Win T., Laroche C.M., Groves A.M., White C., Wells F.C., Ritchie A.J., Tasker A.D. (2004). Use of Quantitative Lung Scintigraphy to Predict Postoperative Pulmonary Function in Lung Cancer Patients Undergoing Lobectomy. Ann. Thorac. Surg..

[33] Yoo I.D., Im J.J., Chung Y.-A., Choi E.K., Oh J.K., Lee S.-H. (2019). Prediction of Postoperative Lung Function in Lung Cancer Patients Using Perfusion Scintigraphy. Acta Radiol..

[34] Moneke I., von Nida C., Senbaklavaci O., Elze M., Meyer P.T., Passlick B., Goetz C., Titze L. (2024). SPECT/CT Accurately Predicts Postoperative Lung Function in Patients with Limited Pulmonary Reserve Undergoing Resection for Lung Cancer. J. Clin. Med..

[35] Jeong Y.H., Lee H., Jang H.J., Park D.W., Choi Y.Y., Lee S.J. (2024). Predicting Postoperative Lung Function Using Ventilation SPECT/CT in Patients with Lung Cancer. J. Thorac. Dis..

[36] Maciąg B., Wojtyś M.E., Waloryszak A., Wójcik N., Pieróg J., Safranow K., Sulikowski T., Grodzki T., Wójcik J. (2025). Scintigraphic Assessment of Pulmonary Flow in Patients After Pneumonectomy. Diagnostics.

[37] Wójcik J., Grodzki T., Sedlaczek A., Kubisa B., Janowski H., Kochanowski L., Alchimowicz J., Pieróg J., Kozak A., Urbański S. (2008). Scintigraphic Assessment of the Pulmonary Perfusion After Pneumonectomy. Pneumo. Info.

[38] Kotloff R.M., Hansen-Flaschen J., Lipson D.A., Tino G., Arcasoy S.M., Alavi A., Kaiser L.R. (2001). Apical Perfusion Fraction as a Predictor of Short-Term Functional Outcome Following Bilateral Lung Volume Reduction Surgery. Chest.

[39] Levitzky M.G. (2013). Pulmonary Physiology: Chapter 4. Blood Flow to the Lung.

[40] Martin C.J., Cline F., Marshall H. (1955). Lobar Alveolar Gas Concentration after Pneumonectomy1. J. Clin. Investig..

[41] Chen W., Xinwei H. (2018). Airway Stenting in Interventional Radiology.

[42] Fernández L.G., Isbell J.M., Jones D.R., Laubach V.E., Cardoso P.F.G. (2012). Compensatory Lung Growth After Pneumonectomy. Topics in Thoracic Surgery.

[43] Ghammad K., Fievez P., Remy P. (2015). Compensatory Lung Growth after Pneumonectomy: Case Report. Acta Chir. Belg.

[44] Paisley D., Bevan L., Choy K.J., Gross C. (2014). The Pneumonectomy Model of Compensatory Lung Growth: Insights into Lung Regeneration. Pharmacol. Ther..

[45] Topaloglu O., Aktepe R., Kilic K.N., Karapolat S., Uzun A.Y., Senturk Topaloglu E., Turkyilmaz A., Ozden S., Gumus A., Tekinbas C. (2025). Morphological and Functional Analysis of Residual Lung After Pneumonectomy in Lung Cancer Surgery via 3D-CT Method. Life.

[46] Thet L.A., Law D.J. (1984). Changes in Cell Number and Lung Morphology during Early Postpneumonectomy Lung Growth. J. Appl. Physiol. Respir. Environ. Exerc. Physiol..

[47] Wilcox B.R., Murray G.F., Friedman M., Pimmel R.L. (1979). The Effects of Early Pneumonectomy on the Remaining Pulmonary Parenchyma. Surgery.

[48] Buhain W.J., Brody J.S. (1973). Compensatory Growth of the Lung Following Pneumonectomy. J. Appl. Physiol..

[49] Werner H.A., Pirie G.E., Nadel H.R., Fleisher A.G., LeBlanc J.G. (1993). Lung Volumes, Mechanics, and Perfusion after Pulmonary Resection in Infancy. J. Thorac. Cardiovasc. Surg..

[50] Mergoni M., Rossi A. (2001). Physiopathology of acute respiratory failure in COPD and asthma. Minerva. Anestesiol..

[51] Shibazaki T., Mori S., Arakawa S., Tsukamoto Y., Nakada T., Takahashi Y., Ohtsuka T. (2024). Compensatory Expansion of the Right Middle Lobe: Volumetric and Functional Analysis of the Changes after Right Upper or Lower Lobectomy. Updates Surg..

[52] Shibazaki T., Mori S., Suyama Y., Arakawa S., Tsukamoto Y., Kato D., Kinoshita T., Nakada T., Ohtsuka T. (2025). Effect of Residual Lung Expansion on Pulmonary Function after Lobectomy. Gen. Thorac. Cardiovasc. Surg..

[53] Cournand A., Riley R.L. (1950). Pulmonary Circulation and Alveolar Ventilation Perfusion Relationships after Pneumonectomy. J. Thorac. Surg..

[54] Taleska G., Trajkovska T., Cardoso P.F.G. (2012). The Effect of One Lung Ventilation on Intrapulmonary Shunt During Different Anesthetic Techniques. Topics in Thoracic Surgery.

[55] Petersson J., Glenny R.W. (2014). Gas Exchange and Ventilation-Perfusion Relationships in the Lung. Eur. Respir. J..

[56] Grodzki T., Alchimowicz J., Kozak A., Kubisa B., Pieróg J., Wójcik J., Bielewicz M., Witkowska D. (2008). Additional Pulmonary Resections after Pneumonectomy: Actual Long-Term Survival and Functional Results. Eur. J. Cardiothorac Surg..

